# *Mucor circinelloides*: a model organism for oleaginous fungi and its potential applications in bioactive lipid production

**DOI:** 10.1186/s12934-022-01758-9

**Published:** 2022-02-28

**Authors:** Abu Bakr Ahmad Fazili, Aabid Manzoor Shah, Xinyi Zan, Tahira Naz, Shaista Nosheen, Yusuf Nazir, Samee Ullah, Huaiyuan Zhang, Yuanda Song

**Affiliations:** 1 Colin Ratledge Center for Microbial Lipids, School of Agricultural Engineering and Food Science, Shandong University of Technology, Zibo, 255000 China; 2 University Institute of Diet and Nutritional Sciences, Faculty of Allied Health Sciences, The University of Lahore, Lahore, 54000 Pakistan; 3 School of Food and Biological Engineering, Jiangsu University, Zhenjiang, 212013 People’s Republic of China; 4 Department of Food Sciences, Faculty of Science and Technology, Universiti Kebangsaan Malaysia, UKM, 43600 Bangi, Selangor Malaysia

**Keywords:** *Mucor circinelloides*, Lipids, Polyunsaturated fatty acids, Metabolic engineering, Carotenoids, Bioremediation

## Abstract

Microbial oils have gained massive attention because of their significant role in industrial applications. Currently plants and animals are the chief sources of medically and nutritionally important fatty acids. However, the ever-increasing global demand for polyunsaturated fatty acids (PUFAs) cannot be met by the existing sources. Therefore microbes, especially fungi, represent an important alternative source of microbial oils being investigated. *Mucor circinelloides*—an oleaginous filamentous fungus, came to the forefront because of its high efficiency in synthesizing and accumulating lipids, like γ-linolenic acid (GLA) in high quantity. Recently, mycelium of *M. circinelloides* has acquired substantial attraction towards it as it has been suggested as a convenient raw material source for the generation of biodiesel via lipid transformation. Although *M. circinelloides* accumulates lipids naturally, metabolic engineering is found to be important for substantial increase in their yields. Both modifications of existing pathways and re-formation of biosynthetic pathways in *M. circinelloides* have shown the potential to improve lipid levels. In this review, recent advances in various important metabolic aspects of *M. circinelloides* have been discussed. Furthermore, the potential applications of *M. circinelloides* in the fields of antioxidants, nutraceuticals, bioremediation, ethanol production, and carotenoids like beta carotene and astaxanthin having significant nutritional value are also deliberated.

**Supplementary Information:**

The online version contains supplementary material available at 10.1186/s12934-022-01758-9.

## Introduction

Fatty acids are an integral part of lipids, comprising of hydrocarbon chains terminating with carboxylic acid groups. Many organisms naturally synthesize fatty acids in high amounts and are attractive sources for the catalytic production of biofuels as well as other industrially important chemicals. Polyunsaturated fatty acids (PUFAs) are vital molecules amongst all fatty acids and are important in maintaining the health of humans [1]. Most important PUFAs are those which are not synthesized by the body and therefore must be consumed through diet [1, 2]. Due to the extensive use of ω-3 PUFAs and ω-6 PUFAs in various fields, particularly food industries and dietary supplements, their production has gained substantial attention in the present times. The traditional sources of PUFAs like plant seeds and fish oils have been limited due to restricted yield and unstable procedures [3, 4]. Currently, lipid biotechnology is emerging as an important division of research due to advancements in analytical techniques, thereby enhancing various applications of lipid and fatty acid by-products in various industries. Though both essential and nonessential PUFAs are found in plant and animal kingdom, microbes mainly fungi and algae are the attractive alternative platforms for their production. The oleaginous microbes can be economically more feasible and sustainable than plants and animals. There have been several investigations regarding diversity of microbial hosts for their fatty acid accumulation potential. Microorganisms usually use simple energy and carbon source, and sometimes they even convert waste into high-value oil [5]. Among microbes, filamentous fungi have been explored for a wide range of macromolecule generation, like enzymes, lipids, structural polymers, and other compounds used in nutraceutical and pharmaceutical industries all over the globe [6]. Yield enhancement under cost-effectiveness is one of the key obstacles for PUFA generation in microbial biotechnology. A better understanding of lipid biochemistry would generate novel prospects to develop endurable microbic PUFAs. From industrial uses to lab-based studies, the advantages of investigating PUFAs in oleaginous fungi, *M. circinelloides,* can be overwhelming.

*Mucor circinelloides* is a model oleaginous fungus for studying lipid accumulation and is considered as an exemplary organism for the generation of fatty acids and other essential bioactive compounds [7–9]. In United Kingdom, it was documented as the first organism for the commercial-scale production of γ-linolenic acid (GLA; 18:3, delta-6, 9, 12) in mid 1990s. [10]. The current strategy of metabolic engineering and culture condition optimizations effectively enhances lipid production and overcomes the traditional limitations of low yield [11]. To increase the potential of lipid production in *M. circinelloides* with a low budget and greater efficiency is the most challenging in overcoming several factors, such as low oleaginicity, low biomass, and slow cell growth. Genetic modification of *M. circinelloides* has been done to produce a variety of biotechnologically important precursors for the functional food and biochemical industries. The composition of fatty acids revealed that it typically contains long-chain fatty acid residues. However, lack of native pathways for many PUFAs production eventually makes it less attractive for versatile industrial applications. Therefore, various tactics have been employed to recompense for this feature in order to meet industrial demands. Optimization of different carbon sources can also be utilized to enhance the lipid accumulation. e.g., recently utilization of soybean oil as carbon source has shown elevation of 43.8% in lipid accumulation [12].

Herein we have provided insights into metabolic engineering of PUFAs in *M. circinelloides* in the generation of recombinant strains to improve the lipid yield. Moreover we have elucidated the genomics, proteomics, metabolic flux analysis, underlying biochemistry of lipid accumulation, metabolic engineering strategies, system biology and various applications of *M. circinelloides* in detail which could be valuable for microbial lipid research in future.

## Characteristics of *M. circinelloides*

**Fig. 1:**
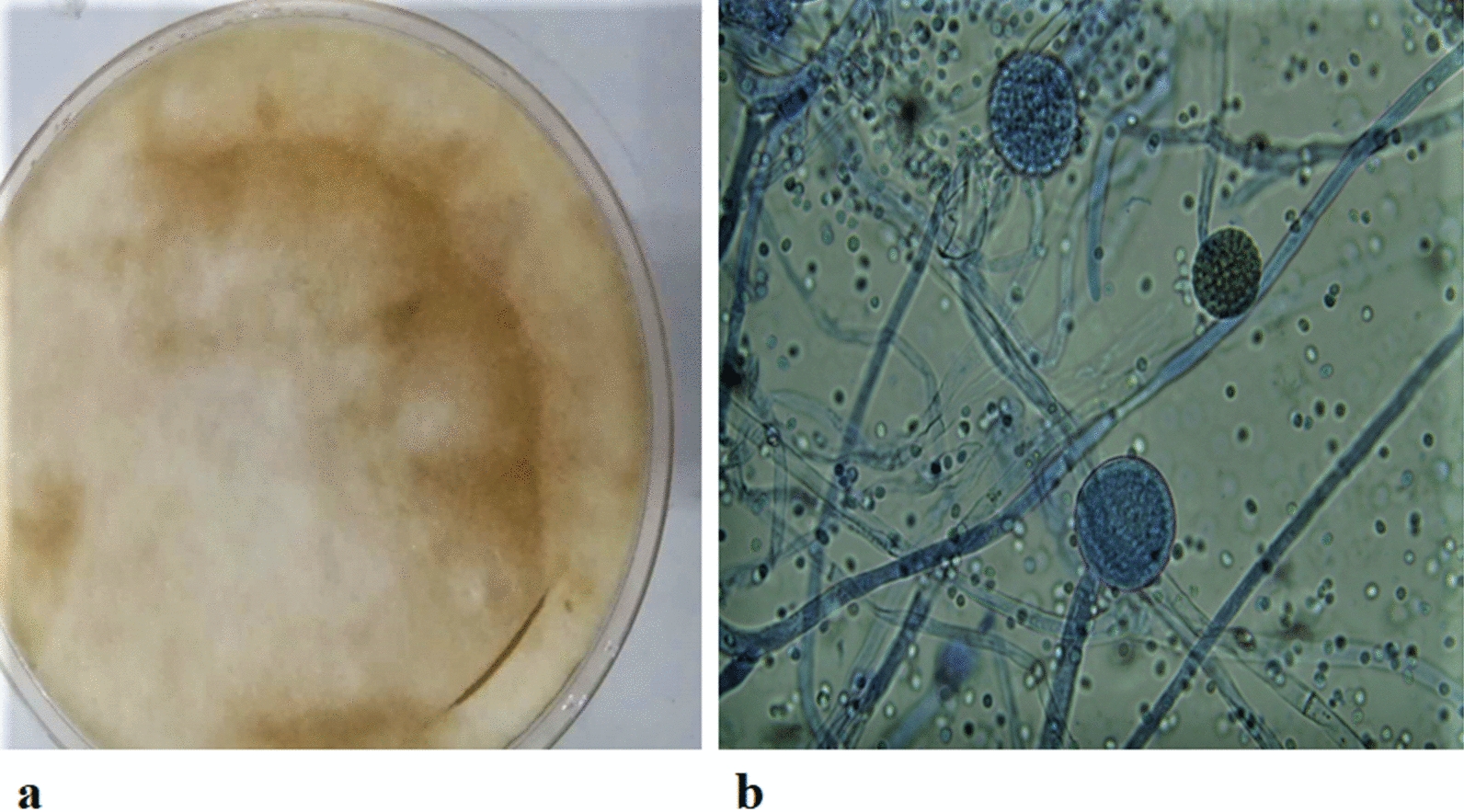
Images of *M. circinelloides* **a** *M. circinelloides* growth pattern on agar plate **b** Microscopic image of lactophenol cotton blue-stained mycelia of *M. circinelloides*

Mucor species are widespread and predominantly saprotrophic [13]. From animal and plant tissues to dung or decayed plant substances, these ubiquitous microorganisms can colonize contrasting and multiple environments. Until now, several hundred possible Mucor species have been found from the initial observation of a Mucor specimen under a microscope in 1665 [7]. *M. circinelloides* is a dimorphic fungus [14], which may occur both in the form of mold and yeast. It belongs to the Kingdom: Fungi, Phylum: Mucoromycota, Order: Mucorales and the Family Mucoraceae. It has a global distribution being mainly present in root vegetables, dung and soil. Growth pattern and microscopic image of *M. circinelloides* is shown in Fig. 1.

*M. circinelloides* are asexually transmitted and have two types of sporangiophores: sympodially branched and elongated [15]. Bigger sporangia which at first are white and gradually change their colour to greenish-brown form part of elongated sporangiophores. They are globular in shape and have a size of 40 to 80 μm; known as 'bobbing heads' [15].

The *M. circinelloides* colonies grow rapidly and reach a height of up to 2 cm [15]. Colony growth is poor and sparse on CYA (Czapek Yeast Autolysate) plates, most frequently spreading around the whole petri dish [16]. These colonies tend to be yellow or light grey, with a colourless reversal and have a diameter of 60 mm or more [16]. The whole malt extract agar petri plate is also filled up by the colonies, creating colours identical to the ones found on plates of CYA [16]. Yeast morphology can also be assumed by *M. circinelloides,* and it has been isolated from normal stool specimens, human urine and frogs in this morphology [15]. The growth and sporulation of *M. circinelloides* is fine at 5–10 degree Celsius [17] and very low at 37 degree Celsius, which is regarded as the upper limit temperature for its growth [18]. *M. circinelloides* can absorb nitrate as well as ethanol [18] and has the minimum water activity of 0.9 for growth. Tall sporangiophores decrease in length and number at lower temperatures. Temperature can affect the sporulation, growth, and presence of short and tall sporangiophores, but it does not affect the size, uniformity and shape of sporangiophores [17].

Comparison of different strains of *M. circinelloides* with other oleaginous microorganisms is provided in Table 1. Different *M. circinelloides* strains have various advantages like (1) availability of distinctive fatty acid profiles, containing fatty acids like GLA that can’t be produced in significant amounts by other oleaginous microorganisms [19, 20]. (2) Cultivation of some strains of this fungi can be done on renewable sources of carbon like agricultural residues [21, 22]. (3) It can be easily genetically modified to produce high amount of lipid [8, 23–27]. (4) Applications of *M. circinelloides* in the fields of carotenoids, nutraceuticals, antioxidation and bioremediation are among its important advantages [9, 28–32]. The disadvantages of certain species of Mucor, including *M. circinelloides* are (1) Causing mucormycosis by acting as animal and human pathogen [7, 33]. (2) Being less cost efficient.

**Table 1:** Comparison of different strains of *M. circinelloides* with other oleaginous microorganisms in terms of lipid content and substrate specificity

Oleaginous microorganism	Strains/Species	Lipid accumulation (%, *w/w*)	Substrate specificity	References
Microalgae	*Botryococcus braunii*	28	Altered Chu 13 photoautotrophic medium	[161]
	*Tetraselmis elliptica*	14	Flory Photoautotrophic medium	[162]
	*Scenedesmus *sp.	34.10	CO2:CH4 40:60 and Altered Chu 13 photoautotrophic medium	[163]
	*Chlamydomonas reinhardtii,* CC1010	59	Photoheterotrophic medium	[164]
Yeast and filamentous fungi	*Rhodotorula glutinis*	20	Glucose and Monosodium glutamate	[165]
	*Cryptococcus* sp. (KCTC 27583)	34	Pretreated peel of banana	[166]
	*Lipomyces starkeyi*	48	Glucose and xylose	[167]
	*Trichosporon fermentans* CICC 1368	36	Vines derived from waste sweet potato that has undergone simultaneous fermentation and saccharification	[168]
	*M. circinelloides* CBS 277.49; W11	15 for CBS 277.49 36 for W11	Glucose	[169]
	*M. circinelloides* ATCC 1216B	13–29	Agricultural residues	[21, 22]
	*M. circinelloides* URM 4182	14.0 (for glycerol) 43.0 (for glucose)	Glycerol and glucose	[170]
	*M. circinelloides* CBS 277.49	52	Co-products of ethanol and corn	[171]
	*M. circinelloides* CCF 127	42.7	Sunflower oil	[172]
Bacteria	*R. opacus* PD630	70 for dextrose 14 for dairy wastewater	Dextrose and dairy wastewater	[173]
	*R. opacus* PD630	46	Pulp from hardwood Kraft	[174]
	*Gordonia *sp*. DG*	40 for maize oil 52 for sunflower oil 13 for olive oil	Maize, sunflower and olive oil	[175]

## Genomics, proteomics, and metabolic networks of *M. circinelloides*

### Genomics

**Fig. 2:**
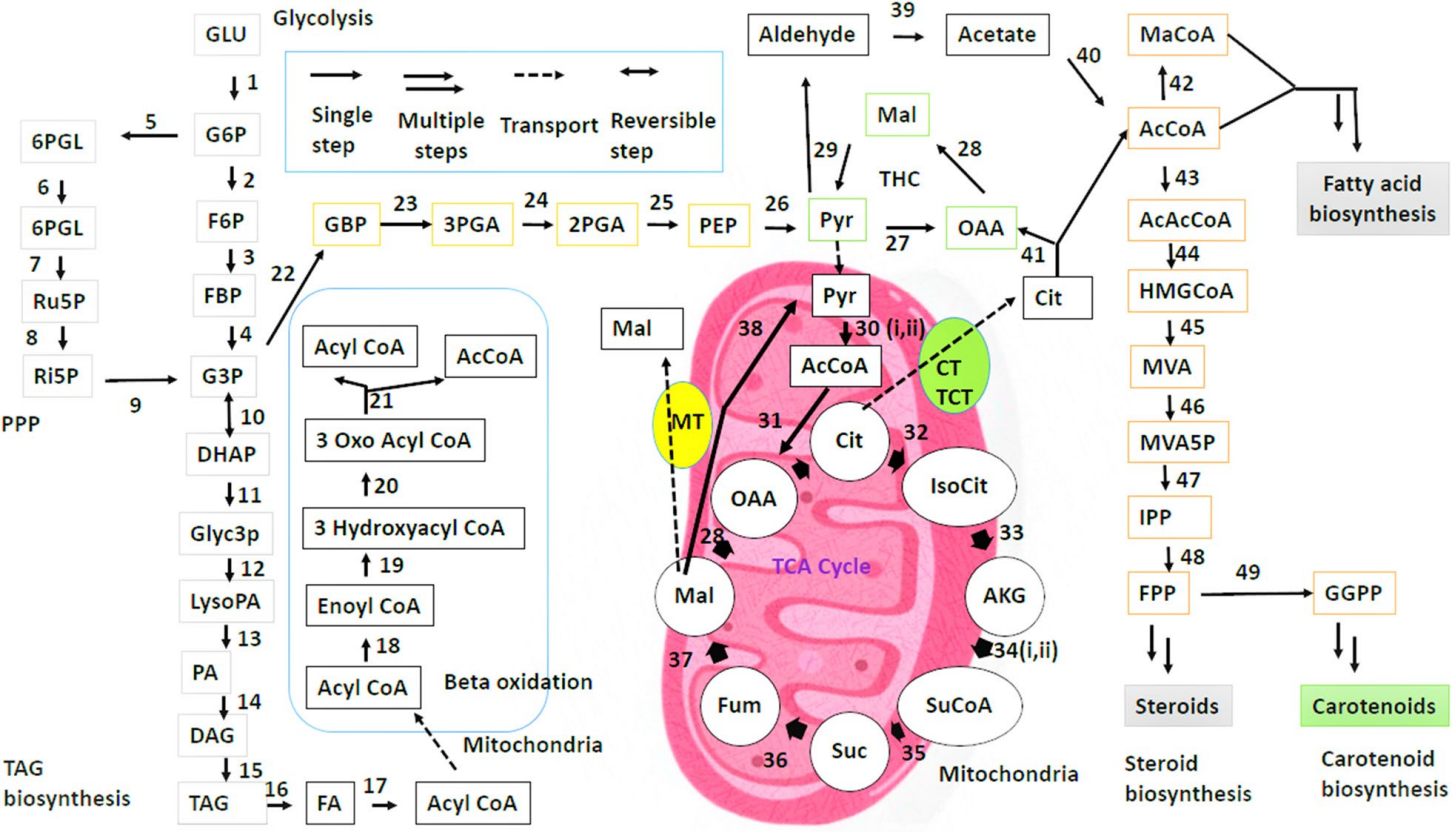
This picture demonstrates different pathways of carbon and lipid metabolism such as glycolysis, pentose phosphate pathway, TAG biosynthesis, transhydrogenase cycle, TCA cycle, fatty acid biosynthesis, fatty acid beta oxidation, carotenoid and steroid biosynthesis. Substrates have been abbreviated and enzymes have been numbered. Abbreviations of substrates, transporters and pathways are provided in Additional file 1: Table S1. List of numbers denoting different enzymes is provide in Additional file 2: Table S2

A strong capability to identify numerous differentially expressed genes among distinct strains of microbes in the identical species is aided by approaches of comparative genomics [34]. Joint Genome Institute sequenced the genome of *M. circinelloides* CBS 277.49—a low lipid generating strain (15% w/w lipid, CDW) and compared it with *M. circinelloides* WJ11—a high lipid generating strain (36% w/w lipid, cell dry weight, CDW). The common characteristics, at the degree of gene identity and gene order, of CBS 277.49 and WJ11 suggested that they are similar. The WJ11 G+C content and size of genome assembly were found to be about thirty-nine percent and 35.4 Mb, respectively. While using the MAUVE program, it has been revealed that the genomes of the two strains have numerous homologous regions which were mostly co-linear. The number of genes for lipid accumulation enzymes was compared via reorganization of lipid metabolism and central carbon routes [35]. For each strain, several distinct protein-coding genes engaged in the growth of cells, lipid and carbohydrate metabolism were determined. Genes encoding different enzymes for the processes of pentose phosphate pathway (PPP) (Fig. 2) and glycolysis (Fig. 2) were found to be similar in both the strains. However, in WJ11 more genes coded for G6PDH compared to CBS 277.49, which might be signifying more NADPH generation for synthesis of fatty acids in case of WJ11. Moreover, compared to other enzymes of PPP and glycolysis hexokinase was found to be encoded by more number of genes. This suggested a significant role of hexokinase genes in more uptake of glucose in *M. circinelloides,* similar to other microorganisms like *Aspergillus niger, Mortierella alpina* etc. [33, 36–39]. Some enzymes of TCA cycle (Fig. 2) were found to be encoded by more genes in CBS 277.49, suggesting TCA cycle to be more dynamic for CBS 277.49 as has been demonstrated in earlier investigations [11]. Enzymes responsible for TAG, steroid and phospholipid biosynthesis were found to be coded by similar gene numbers. However, phosphatidate cytidylyltransferase and glycerol-3-phosphate dehydrogenase were coded by less and more number genes respectively in WJ11 strain. Glycerol flux during triacylglycerol biosynthesis might be affected by these variations. Identification of genes encoding enzymes for fatty acid synthesis and fatty acid beta oxidation was also done, suggesting active fatty acid synthesis and beta oxidation process. There were many unique genes reported to be involved distinctly in carbohydrate and lipid metabolism in WJ11 and CBS277.49. These unique genes were suggested to be responsible for discrete lipid accumulation patterns among the two strains For example G6PDH encoded by unique gene n WJ11 was suggested to be responsible for additional NADPH generation for accumulation of lipids. Taken together, the comparative analysis of genomes of the two strains and identification of unique genes lays a strong foundation for future direction of research in lipid metabolism.

### Proteomics

Accumulation of lipids is a complex procedure affecting numerous pathways of metabolism; therefore, it is extremely hard to attain optimum lipid output by manipulating few genes. Therefore, to gain insights into the molecular mechanism of lipid accumulation, exploration of lipid metabolic rate at different phases is needed. Although substitute strategies exist to comprehend the underlying molecular mechanisms of nitrogen-deficiency induced cellular reactions, proteomics of microbes is becoming an effective opportunity for exploring intricate processes of the cell. Additionally, the new utility of products from different genes can also be figured out since proteomics reflect the post-translational changes as well as the rate of translation. In order to figure out the lipid accumulation systems, it is essential to recognize proteins that are down/up-regulated under deficiency of nitrogen. Therefore, to gain an understanding of lipid metabolism in microalgae and yeast under nitrogen-limited conditions, comparative proteomics was probed [40–43].

To get novel insights into lipid metabolic mechanism in *M. circinelloides* WJ11, Tang et al. [44] compared proteomes at balanced growth phase, the gradual lipid accumulation phase, and quick lipid accumulation phase. Analysis of proteomics evidenced that nitrogen limitation reduced the amino acid metabolism but enhanced the glutamine synthetase expression associated with assimilation of ammonia for the cellular nitrogen supply. After deficiency of nitrogen, there was a down-regulation of proteins active in the TCA cycle like succinate dehydrogenase, isocitrate dehydrogenase, fumarate hydratase, and succinyl CoA ligase. At the same time, there was up-regulation of numerous proteins required for glycolytic pathways like pyruvate kinase, glyceraldehyde-3-phosphate dehydrogenase, fructose bisphosphate aldolase, and enolase, indicating a much better flow of carbon into the biosynthesis of fatty acids. Additionally, there was an elevation in NADPH generation, triggered by up regulation of enzymes of the PPP like transketolase, transaldolase, and glucose-6-phosphate dehydrogenase. Moreover, there was a cellular reaction caused by nitrogen-limited conditions in the form of down regulation of the metabolism of proteins and nucleic acids and up-regulation of several proteins engaged with redox homeostasis, molecular chaperone, signal transduction, and energy metabolism. Upon nitrogen deficiency, more NADPH for the biosynthesis of fatty acids along with greater carbon flow to acetyl-CoA was provided by organized regulation of central carbon metabolism [44].

### Metabolic networks

*Mucor circinelloides’s* genome-scale metabolic network introduced for the first time by Vongsangnak et al. [45] exhibited coordination between lipid accumulation, cell growth, and overall metabolizing processes. To construct this model, Vongsangnak et al. [46] utilized prediction tools along with a database of different pathways and proteins. Phenotypic Phase Plane Analysis and Flux Balance Analysis were then employed to substantiate this model. Further, they used Flux Variability Analysis and comparative tools to investigate phenotypic and metabolic traits among relevant oil-producing microorganisms like *Y. lipolytica, M. circinelloides,* and *M. alpina*. Their results reported that *M. circinelloides* had good adaptability for utilization of nutrients because its genome-scale model revealed that it contained several genes for lipid, carbohydrate, and amino acid metabolisms [46]. When the genome-scale metabolic models of three oleaginous microorganisms were compared, it was noted that *M. circinelloides* had more genes for lipid, carbohydrate and amino acid metabolism. The presence of more number of genes encoding for hydrolytic enzymes in carbohydrate metabolism explained the use of wide range of carbon sources like sucrose, maltose, lactose starch and raffinose by *M. circinelloides*. On the other hand *Y. lipolytica* and *M. alpina* are reported to be poor utilizers of variety of carbon sources [47, 48].

Vongsangnak et al. [49] employed omic scale analysis to comprehend the metabolizing performance of *M*. *circinelloides* WJ11. Metabolic network standard was further improved by the addition of protein and gene expression records. Their results revealed involvement of several genes in nitrogen, lipid, central carbon, and amino acid metabolism. Protein domain analysis was employed to recognize distinctive characters of the biosynthetic route in carotenoids. Supplementary routes for the metabolism of lipid and fatty acid synthesis precursors like fatty acyl substrate, NADPH, and acetyl-CoA were indicated with the help of systematic compilations. Fascinatingly, it was reported that the metabolism of amino acids played an important part in the response system of cells for oleaginous microbes during the imbalance of nutrients via lipogenesis. Integration of data for the expression of genes with networks of metabolism helped in elucidating the collaborative role of some enzymes responsible for the degradation of lipids. In conclusion, their investigations gave a tangible insight into system biology for the production of lipids in oil generating microbes that can be useful for industrial biotechnology [49].

## Biochemistry of lipid accumulation

With the exhaustion of nitrogen or phosphate from the medium, lipid accumulation in oleaginous microorganisms gets initiated. When nitrogen is depleted in the medium, the remaining glucose persists to be taken up by the cells and transformed into triacylglycerols at more or less the same rate at which lipids were synthesized during the balanced growth process. The nitrogen supply limitation implies that cell proliferation is averted; therefore, the cells start storing the lipids and cannot proliferate anymore, and hence we have the oleaginous microorganism! [50]. Non-oleaginous microbial organisms are defined as those organisms that cannot store lipids. When they are placed in a nitrogen-deficient environment, they either stop cell division or, in other cases, redirect the carbohydrates into various polysaccharide formations, like mannans, glucans, glycogen, etc. They can only accumulate oil to less than 10% [51].

Therefore, it can be inferred that as this biological mechanism is similar to all microbes, the capacity of a microorganism to accumulate high amount of lipids must stand beyond the instant region of fatty acid generation. Two reasons could explain for oleaginicity: the capability of a microbe to endlessly generate acetyl-CoA into the cell cytosol (it is the precursor of fatty acid synthase pathway) and its capability to generate a sufficient amount of NADPH as an important reducting agent used in the biosynthesis of fatty acids. Existence of ATP: citrate lyase (ACL, reaction no. 1) [50] has been linked to the production of acetyl-CoA in oleaginous microbial species, but it doesn't exist in most non-oleaginous bacteria:

1 $${\text{CoA }} + {\text{ Citrate }} + {\text{ ATP}} \to {\text{Oxaloacetate }} + {\text{ acetyl }} - {\text{ CoA }} + {\text{ ADP }} + Pi$$

For optimal functioning of ACL, its substrate (citric acid) must access the cell cytosol where fatty acid biosynthesis takes place. Of course, citric acid is synthesized within the mitochondrion of the cell as a component of the tricarboxylic acid (TCA) cycle. A peculiar aspect of oleaginous microorganisms that enables the accumulation of citric acid is the dependence of isocitrate dehydrogenase activity on AMP in the TCA cycle. In contrast, no such dependence occurs in the enzymes of non-oleaginous microbes. The deaminase activity of the AMP deaminase (AMPD) mediates the AMP concentration in the cell, as seen in reaction no. 2 [51]:

2 $${\text{AMP}} \to {\text{NH}}_{{3}} + {\text{inosine 5}}^\prime \, - {\text{monophosphate}}$$

AMPD's function, likely as a means of scavenging auxiliary ammonium ions from intracellular materials, is overexpressed by the onset of limitation of nitrogen in the surroundings of the oleaginous microbial species. Under nitrogen limitation, generation of acetyl-CoA occurs as shown in reaction 1. Oil generating cells display an elevated level of AMPD at the onset of nitrogen exhaustion that is almost five times higher in cells before the restriction of nitrogen. The level of AMP inside the cells, along with the mitochondrial content, gets reduced upon elevation in the activity of AMPD. Since isocitrate dehydrogenase is essentially dependent on the activity of AMP in oleaginous cells, it is thus prevented from functioning as a result of a reduced level of AMP. Therefore, isocitrate gets accumulated as it is not metabolized. Via aconitase, isocitrate is then balanced with citric acid, thereby resulting in accumulation of citrate inside mitochondrion. The mitochondrial membrane has an important citrate efflux mechanism for transporting citrate in exchange for malate. Citrate reaches the cytosol and is acted upon by ACL in order to deliver oxaloacetate and acetyl-CoA. This acetyl-CoA is then used during the synthesis of fatty acids. The malate dehydrogenase converts the oxaloacetate into malate used in the citrate efflux system as a counter ion [51]. Figure 2 gives an overview of carbon and lipid metabolism, showing different steps that take place during the process.

Although under nitrogen-limited conditions, this synthesis of acetyl-CoA from glucose can account for the influx of carbon atoms via the fatty acid synthase (FAS) pathway, still the tale is incomplete. Such cells have been identified that cannot accumulate lipids, even though they have ACL activity. But the corollary is false: a lipid-accumulating microorganism that does not have ACL activity is yet to be discovered. Therefore, various other enzymes must exist to ascertain the accumulation of lipids.

It’s noteworthy that, NADPH is necessary to accomplish the synthesis of fatty acids since they are profoundly reduced constituents. Sixteen moles of NADPH are required for the synthesis of one mole of fatty acid having a chain length of eighteen carbon atoms as two moles of NADPH are required to hydrogenate each 3-keto-fatty acyl group resulting from each acetyl-CoA condensation reaction with malonyl-CoA as a component of the fatty acid synthetase enzyme complex into the saturated acyl fatty acid chain which is further subjected to a process of chain-lengthening [51].

For the biosynthesis of fatty acids, malic enzyme (reaction no. 3) is suggested to be an important source of NADPH [51]:

3 $${\text{NADP}}^{ + } + {\text{ Malate}} \to {\text{NADPH }} + {\text{ Pyruvate }} + {\text{ CO}}_{{2}}$$

From this information, the identification of genes that code for the key enzymes involved in the accumulation and production of fatty acids should be a rational step. A summary of enzymes and genes engineered over the years to boost the generation of lipids in *M. circinelloides* is presented in Table 2.

**Table 2:** A summary of metabolic engineering of different enzymes and genes in *M. circinelloides* to boost the production of lipids

Strain used *M. circinelloides*	Research specificity	Effect on lipid percentage	References
CBS 108.16	Malic enzyme recombinant	2.5 fold increase in lipid content	[8]
CBS 277.49	Malic enzyme recombinant	No increase in lipid content	[57]
CBS 277.49	*6pgd* and *g6pd* recombinant	20–30% increase in lipid content	[66]
WJ11	*leuB*	67–73% increase in lipid content	[23]
WJ11	*g6pdh1, g6pdh2*	In case of *g6pdh1* total fatty acid content was elevated by 23–28% and for *g6pdh2* it was elevated by 42–47%	[23]
CBS277.49, CBS108.16 and WJ11	Xylose Isomerase and Xylulokinase	Lipid content is elevated slightly	[67]
WJ11 and CBS 277.49	Citrate transporter	44% lipid content elevation	[24]
WJ11 and CBS 277.49	Tricarboxylate transporter	68% lipid content elevation	[24]
CBS277.49	Malate transporter	70% lipid content elevation	[25]
WJ11	Snf-β recombinant	32% lipid content elevation	[79]
CBS 277.49	Lipases: Lip6 and Lip10	Lip6: enhanced lipid accumulation by 9–24% Lip 10: enhanced lipid accumulation by 14%	[26, 27]
CBS 277.49	Diacylglycerol acyltransferase	Might enhance lipid accumulation	[93]

## Central carbon flux distribution during lipid accumulation

^13^C-labeled metabolism flux investigation via intracellular flux assessment based on stable isotopes was carried out for the first time by our group to thoroughly understand the lipid accumulation mechanism in *M. circinelloides* WJ11 [52]. Results concluded that in the WJ11 strain, PPP plays a vital role in NADPH generation during the biosynthesis of lipids. Flux data analysis showed that glycolysis was down-regulated while PPP was up-regulated, which would lead to increased NADPH production. In addition, the TCA cycle was down-regulated, which resulted in increased acetyl-CoA generation. The distribution between different pathways in WJ11 and CBS 277.49 for NADPH consumption and NADPH yield during lipid accumulation (N limited medium or high N medium) is shown in Table 3. Metabolic flux analysis has also been utilized for malate transporter manipulated strain which has been elucidated in detail under malate transporter heading in this review.

**Table 3:** Total NADPH percentage, NADPH Yield, and Consumption percentage in *M. circinelloides* WJ11 and CBS 277.49 during balanced growth phase at low and high nitrogen concentrations

Strain used *M. circinelloides*	Nitrogen Conc	Total NADPH	NADPH yield percentage (%)			NADPH consumption percentage (%)			References
			PPP	ME	ICDH	Lipid	Protein	Nucleic acid	
WJ11	High Nitrogen	1.532	43.60	6.59	49.80	10.78	83.73	5.49	
	Low Nitrogen	1.564	60.64	12.73	28.72	30.08	67.54	2.37	[52]
CBS 277.49	High Nitrogen	1.64	42.74	1.28	55.98	8.06	87.35	4.58	
	Low Nitrogen	1.586	50.31	7.12	42.43	16.05	81.30	2.65	

PPP, ME, and ICDH represent the pentose phosphate pathway, malic enzyme, and isocitrate dehydrogenase, respectively

## Engineering of different enzymes/genes/pathways to enhance lipid content in *M. circinelloides*

### Malic enzyme

Malic enzyme (ME; EC 1. 1 0.1. 40) is considered to be one of the crucial enzymes for lipid accumulation. It performs decarboxylation irreversibly between Pyruvate and Malate together with the conversion of NADP^+^ into NADPH (reaction 3)_._ It had been suggested previously that this particular NADPH is essential in fatty acid biosynthesis. No other enzyme-generating NADPH seemed to be in a position for offering the requisite reducing strength in fatty acid synthase function [53, 54]. Utilizing *M. circinelloides* as a model organism, it has been found that sesamol, as a particular inhibitor during the operation of ME activity, reduces the build-up of lipids from twenty-five percent of the cellular biomass to two percent without negatively impacting growth [53].

Five putative genes that encode for MEs have been identified from relative protein-rich sequence analysis along with a purposeful project of the genome of *M. circinelloides* (http://genome.jgipsf.org/Mucci2/Mucci2.home.html). These five genes synthesize five proteins in total, three in mitochondria and two in the cytosol [55]. Among the genes encoding MEs in Cytosol, gene ID 182779 encodes 2 isoforms, namely IV and III linked with lipid accumulation [56]. The role of ME isoforms III/IV in the accumulation of lipids is further substantiated when these genes were overexpressed, causing 2.5 fold expansion in *M. circinelloides* lipid build up [8].

Zhang et al. [8] determined the gene encoding ME isoforms III/IV, and re-established it within *M. circinelloides CBS 108.16* by using a constitutive promoter. They achieved a more significant expression of the ME activity and enhanced lipids (almost from 12% of the biomass to 30%). The concept that the rate-limiting phase of biosynthesis of fatty acid is the NADPH furnished by ME was substantially reinforced by this particular observation. Nevertheless, overexpressed strains in this experiment were not stable and viable at the industrial level because the overexpressing genes had been inserted into self-replicating plasmids.

Rodríguez-Frómeta et al. [57] attempted to produce such strains that overexpress the ME gene and are stable as well. With an aim of utilizing such a strain for the generation of biomass ideal for biodiesel transformation, another approach of replacing genes was used to avoid the synthesis of carotenoids inside the cells as they cannot be converted into biodiesel [58]. Intriguingly, the genetically modified strain in the experiment of Rodríguez-Frómeta et al. [57] showed lipid content similar to that of control strain, indicating that other restricting stages in the synthesis of fatty acid pathway might be present as a result of dismissal of the bottleneck based on ME. Investigations carried out by Tang et al. [59] and our group [52] have demonstrated that no doubt, ME plays a role in fatty acid synthesis but it is not the main NADPH generating enzyme in *M. circinelloides*. Overexpression of ME has been found to enhance lipid accumulation in bacterial strains like *Rhodococcus josti* [60]. Effect of ME for enhancement of lipid accumulation has been also found in various microalgae specied like *P. tricornutum, C. pyrenoidosa, Nannochloropsis salina* and *C. protothecoides* [61–64]*.*

### Pentose phosphate pathway

In eukaryotic oleaginous microorganisms, the biosynthesis of lipids occurs in cytosol. This process has significant functions, including furnishing of NADPH and supply of vital progenitors of essential fatty acid residues in the form of acetyl CoA [65]. By means of stoichiometric evaluation, Ratledge [65] measured the sources of NADPH and recommended that ME performs a crucial function in the majority of organisms but cannot deliver the total NADPH required for the biosynthesis of lipids, suggesting requirement of additional NADPH sources. Although there is a possibility that cytosolic isocitrate dehydrogenase reaction may generate several NADPH molecules, PPP reactions seem to be the likely pathway [65]. 6 phosphogluconate dehydrogenase (6PGD; EC: 1.1.1.44) and glucose 6 phosphate dehydrogenase (G6PD; EC: 1.1.1.49) are the enzymes of PPP that are usually regarded as significant sources of NADPH. These enzymes are found to be associated with NADPH-generating reactions catalyzing the transformation of glucose-6-phosphate to ribulose 5 phosphate -a precursor of essential molecules like nucleic acids.

4 $${\text{NADP}}^{ + } + {\text{Glucose-}} {6} {\text{-Phosphate}} \to {\text{NADPH }} + { 6} {\text{-Phospho-}} {\textsc{d}} {\text{-Glucono-}} {1},{\text{5 Lactone}}$$

5 $${\text{NADP}}^{ + } + { 6} {\text{-Phosphogluconate}} \to {\text{NADPH }} + {\text{ CO}}_{{2}} + {\text{ Ribulose 5-}} {\text{Phosphate}}$$

mRNA levels and activities of 6PGD and G6PD were found to be substantially improved in *M. circinelloides* strain CBS 277.49 overexpressing 6PGD and G6PD genes [66]. In comparison to the control strain, elevated mRNA levels, as well as elevated activities of 6PGD and G6PD, contributed to a 20 to 30 percent increase in the lipid content of cells in mutated strains. These findings indicated that the essential NADPH suppliers could be 6PGD and G6PD from the PPP and play a crucial part in accumulating lipids in *M. circinelloides* [66].

Tang et al. [23] studied the effect of *leuB, g6pdh2, and g6pdh1* genes on the accumulation of lipids in *M. circinelloides* WJ11. Their investigations revealed that g6pdh2 had a more significant effect on the fatty acid content [23]. PPP has also been found to be attached with assimilation of xylose and the rate of conversion from xylose to lipids can be impacted by its regulation [67]. Chu et al.[67] observed that the overexpression of xylose isomerase and xylulokinase genes enhanced the lipid yield. G6PD and 6PGD are found to be associated with xylose catabolism, and during the study, their activity was estimated via measuring the rate of NADPH production [67]. In another study, when xylulokinase or xylose isomerase gene was overexpressed, lipid accumulation was increased by 8 to 28%. During this study xylose and glucose were fermented together [68].

### Citrate transporter system

#### Malate transporter

Preliminarily, knockout and overexpression of malate transporter (*mt*) gene was helpful in discovering its role in the accumulation of lipids in *M. circinelloides* [69]. It was found that in *mt-*overexpression and *mt*-knockout strains, fatty acid content was increased and decreased by 70% and 27%, respectively [70]. Moreover, knockout of plasma membrane malate transporters in WJ11 strain elevated the lipid content by 10–40% [71].

Wang et al. [25] utilized ^13^C-labeled metabolic flux analysis to unfold the malate transporter regulatory mechanism involved in accumulating lipids. During their investigations, they utilized intrinsic fluxes of metabolism of genetically modified strains of *M. circinelloides*. The assessment of the metabolic flux distribution indicated that the TCA cycle flux ratio of *mt* overexpressed microbial strain was reduced. Subsequently, the strains knocked out for *mt* displayed a reverse phenomenon, exhibiting increased TCA cycle flux ratios relative to the control strain. Besides, in *mt* overexpression strains, although no major impact was observed on the flux rate of ME among the control and genetically engineered strains, a substantially high flux ratio of the PPP was achieved. Together these findings showed that the PPP could be an essential player in providing NADPH [25].

#### Citrate transporter

By catalyzing the transfer of citrate via non-permeable barrier of mitochondrial cristae, the mitochondrial citrate transporter serves as a link between cytosol and mitochondria [72]. During cell development, glucose is converted into pyruvate in glycolysis, which is then transferred inside the mitochondria. Inside the mitochondria, pyruvate is acted upon by pyruvate dehydrogenase and converted into acetyl-CoA. Acetyl-CoA enters into the TCA cycle leading to the formation of citrate by reacting with oxaloacetate (OAA). Hence by undergoing full oxidation, it acts as the main ATP supply for the cell [73]. The TCA cycle is slowed down following an environmental stimulus such as nitrogen restriction, thereby accumulating citrate in mitochondria. Thereafter citrate is shifted from the mitochondria into the cytosol by the transporters, ACL splits the cytosolic citrate into acetyl-CoA and OAA. OAA is reduced to malate, which is translocated back into the mitochondria, while Acetyl-CoA acts as a vital precursor for the biosynthesis of sterols and fatty acids. Alternatively, malate can be converted into pyruvate when acted upon by ME, thereby producing cytosolic NADPH for the biosynthesis of fatty acids and sterols [55, 57, 65]. Citrate functions as an essential regulatory factor for fatty acid synthesis, glycolysis, and gluconeogenesis. Therefore, in oleaginous microorganisms, citrate transport among cytosol and mitochondria that occurs via mitochondrial citrate transporter is vital [65].

The genomes of WJ11 and CBS277.49 were studied by Yang et al. [24], who detected the possible genes involved in the mitochondrial citrate transport system. In the genome of WJ11, five possible genes were discovered. 1 tricarboxylate carrier (TCT), 2 CT (Citrate Transport) Protein, 2 oxoglutarate antiporters, namely SoDIT-b and SoDIT-a, and 1 MT were found to be encoded by these genes. However, except for the inclusion of one SoDIT, the genome of CBS 277.49 contained the same set of genes. WJ11 and CBS 227.49 had similar protein properties. Moreover, the evolutionary relationship of these proteins was identified by phylogenetic analysis. Besides, the expression of these genes was analyzed to anticipate their potential roles in *M. Circinelloides's* lipid metabolism [74]. Citrate transporters' role in modulating biosynthesis of lipids was studied by Yang et al. [24], who over-expressed the WJ11 citrate transporters into CBS 277.49. Their findings revealed that citrate transporter overexpression resulted in increased accumulation of lipids by 45%, whereas tricarboxylate transporter overexpression enhanced lipid accumulation by 68%. It was also found that in citrate transporter and tricarboxylate transporter overexpressing strains, the concentration of extracellular citrate dropped by 20% and 47%, respectively, relative to the control strains. Besides, overexpression of citrate transporter genes triggered downstream steps such as fatty acid synthases and ATP citrate lyase during lipid accumulation, suggesting a higher carbon flow in the biosynthesis of fatty acids [24].

In WJ11, ^14^C-labelled investigation for transportation of citrate was utilized to study mitochondrial citrate transporting mechanism [75]. It was found that transportation of citrate is facilitated by molecules like glutamate, oxaloacetate, isocitrate, malate, oxoadipate, fumarate, aconitate, and succinate. Lipid accumulation in *M. circinelloides* was increased because of enhanced mitochondrial transportation of citrate, which was elevated because of overexpression of *ct* [75].

### AMPK

Essential metabolizing regulators include AMP-activated protein kinase (AMPK) family. AMPKs have a vital function during the metabolism of lipids. Upon stimulation, they inhibit and phosphorylate the activity of acetyl-CoA carboxylase, thus hindering the formation of malonyl-CoA from acetyl-CoA [76]. Carnitine Palmitoyl Transferase, which transports fatty acids for the purpose of their oxidation into mitochondria, is also inhibited by malonyl-CoA. As a result, a low Malonyl-CoA level would eventually increase fatty acid oxidation [77]. Hence, AMPK facilitates the oxidation of fatty acids as well as prevents their synthesis.

During the investigations done by our group [78], the homologs genes of AMPK were studied in CBS277.49 and WJ11 strains. We performed the bioinformatics and transcriptional analysis of genes of AMPK during the process of active growth of strains. These analyses were also performed during the storage of lipids. 2 genes for alpha subunit, 1 for beta subunit, and 6 for gamma subunit of AMPK genes were determined and annotated. Bioinformatics research established the existence of typically preserved domains in these genes. During the lipid accumulation process, the AMPK gene expression level was elevated in CBS 277.49 strain, while a contrasting expression profile was observed in WJ11 strain. Our research provided important insights into AMPK gene interactions with transcriptional lipid metabolism, which may be explored for further studies [78]. During further experiments, our group [79] found 32% elevation in lipid accumulation of Snf-β knockout strain.

## Metabolism of TAG

The microbial TAGs content determines the potential of biodiesel production by an organism. Synthesis of TAGs takes place in two steps: in the first step, glycerol 3-phosphate is acted upon by enzymes phosphate: acyl-CoA acyltransferase and lysophosphatidic acid: acyl-CoA acyltransferase, thereby generating phosphatidic acid [80] which is converted to diacylglycerol (DAG) with the activity of phosphatidate phosphohydrolase. DAG is then catalyzed by diacylglycerol: acyl-CoA acyltransferase and subsequently converted into TAG.

Jackson et al. [81] characterized *M. circinelloides* TAG assembly capable microsomal membrane preparations and reported biochemical features of enzymes involved in acylation. During their study, the main lipids labelled in ^14^C acetate incubated cultures included phosphatidylcholine, phosphatidylethanolamine, and TAG. It was found that label proportion was elevated in TAG and reduced in phospholipids. Moreover, the microsomal membrane section had the highest TAG-synthesizing ability, accumulating enhanced phosphatidic acid concentration. Dynamic in vitro microsomal membranes creation competent of TAG assembly into biosynthetic routes can be useful in comprehending how oils are assembled in potential applications of transgenics [81]. In following sections we have discussed about lipases and DGAT involved in TAG metabolism.

### Lipases

Ester bonds between glycerol and fatty acid residues are hydrolyzed by enzymes known as triacylglycerol hydrolases or lipases (EC 3.1.1.3) [82]. Lipases can hydrolyze TAGs to fatty acids, glycerol, diglycerides, and monoglycerides [83]. Ester synthesis by inter-esterification and esterification as well as trans-esterification reactions can also be catalyzed by lipases. Furthermore, they might be utilized in several biotransformation reactions, including aminolysis, hydrolysis, and synthesis of thioester and amide [84]. As a result of these distinct qualities, lipases are popular in waste treatment [85], fine chemical substances refinery [86], citric acid production [87], and pharmaceutical industries [88].

Fungal cells are viewed as vital sources of lipases [89, 90]. *M. circinelloides* grows well in culture-containing triacylglycerol, indicating a potential source of extracellular lipases [89]. Many scientific studies from a hereditary perspective have ascertained that *M. circinelloides* produces many lipase genes [35, 46, 91]*.*

All lipase coding genes in a high lipid producing strain—*M. circinelloides* WJ11 have been analyzed and compared with the lipase genes in *M. circinelloides* CBS 277.49—a low lipid producing strain by our group [92]. We have also examined the characteristics, such as expression profiling of lipase genes whilst progression and aggregation of lipids, and also their subcellular location and phylogenetic analysis. Our findings showed that many of the proteins contained the usual GXSXG lipase motif, which is classified into four types: alpha/beta-hydrolase III, alpha/beta-hydrolase I, GDSL, and class 3. Interestingly, several lipases were also found to have a distinct H-(X)-4-D acyltransferase motif that could play twin roles in the metabolism of lipids, catalyzing transacylation as well lipid hydrolysis reactions. Based upon the functions of lipases in yeast as well as gene expression profiling of lipases within *M. circinelloides*, it was assumed that many lipases could have been engaged via beta-oxidation, phospholipid synthesis, and degradation of TAG [92].

Fungal lipase- Lip6's role in the accumulation of lipids was reported in 2018 by our group [26]. We reported that in *M. circinelloides,* Lip6 has a versatile enzyme activity in the form of acyltransferase and lipase. To investigate its dual function, site-directed mutagenesis was performed in acyltransferase and lipase motifs of Lip6. Upon loss of lipolysis activity because of mutation in lipase motif whereas no mutation in acyltransferase motif, lipid accumulation elevated by 9–24% and cell biomass by 12–18%. On the other hand, mutation of the acyltransferase motif led to the degradation of lipid [26].

Our group also reported another dual-functional lipase in *M. circinelloides* called Lip 10 [27]. Lip 10 was found to have the trivial activity of lipolysis and a more important phospholipid: DAG acyltransferase (PDAT) activity. Like Lip6, Lip 10 also has lipase and acyltransferase motifs. Lip10's Lipase motif over-expression led to the degradation of lipids. On the other hand, over-expression of its acyltransferase motif enhanced the growth of cells by 12% and generation of lipids by 14%, respectively, relative to the control strain [27].

### DGAT

In many microorganisms, there have been several scientific investigations regarding the role played by Acyl CoA: Diacylglycerol Acyltransferase (DGAT) in the synthesis of TAG. A terminal acylation reaction in the TAG biosynthesis pathway is catalyzed by DGAT, which converts DAG to TAG. Four putative DGATs, McDGAT2B, McDGAT2A, McDGAT1B, and McDGAT1A are found to be present in *M. circinelloides*. These DGATs are classified into subfamilies named DGAT2 and DGAT1. To identify and characterize DGATs present in *M. circinelloides,* Zhang et al. [93] expressed these four genes separately in TAG lacking quadruple mutant of *Saccharomyces cerevisiae* known as H1246. Biosynthesis of TAG was solely restored by McDGAT2B expression, and the content of TAG was significantly elevated in McDGAT2B expression mutants than in mutated strain of *S. cerevisiae* with endogenous expression of DGA1. These experiments suggested that McDGAT2B is vital for the accumulation of TAG, suggesting that it might be a crucial metabolic engineering goal to boost the accumulation of lipids in oleaginous fungi [93].

## Metabolic engineering of *M. circinelloides* for the production of bioactive fatty acids

### PUFA synthesis and metabolic engineering of native and non-native PUFAs

**Fig. 3:**
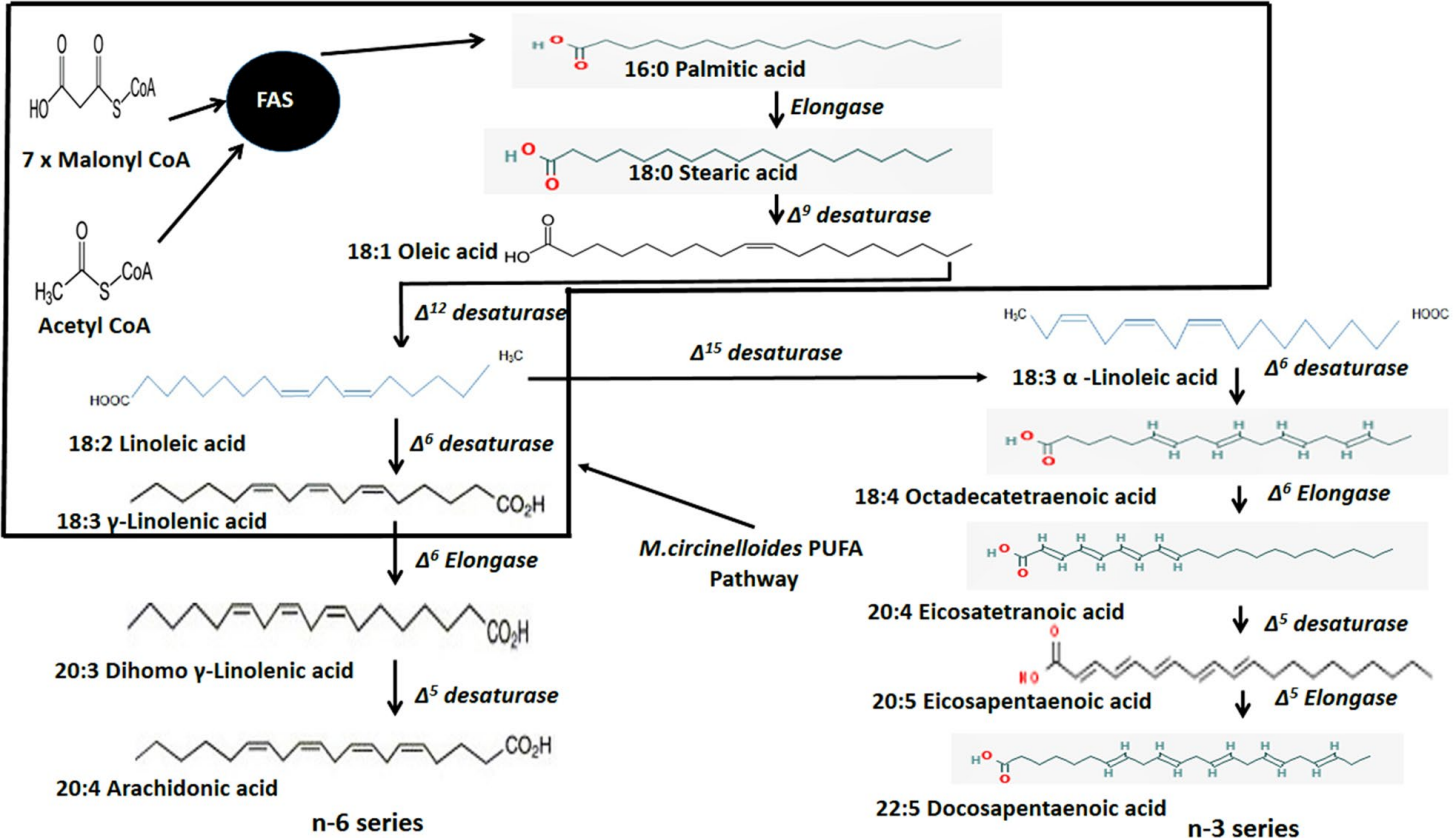
PUFA formation pathways in microorganisms, based on traditional fatty acid synthase pathway. Fatty acid residues are synthesized by the FAS-enzyme complex from malonyl-CoA and acetyl-CoA. PUFAs are categorized into two groups, named as n-3 and n-6 series, depending on the location of the double bond with respect to the terminal -CH_3_ group. PUFA pathway in *M. circinelloides* has been highlighted

Oils derived from microbes, also called single-cell oil (SCO) have been considered as an excellent natural source of lipids, such as PUFAs, rarely found in animal or plant-derived products [94]. *M. circinelloides* produces a large quantity of PUFAs, particularly GLA. It acts as a model organism substantiating fatty acid biochemistry studies. In almost all species, the biosynthesis of fatty acids culminates into saturated fatty acid production with a chain length of either sixteen or eighteen carbons. Through a series of desaturase and elongase enzymes, such fatty acid residues are tailored to create a large number of PUFAs along with unsaturated fatty acids (Fig. 3). PUFAs are, in fact, produced by enzymatic desaturation consisting of oxygenation [95]. In some prokaryotic and eukaryotic organisms, new PUFA biosynthetic pathways have been identified based on polyketide synthesis rather than desaturase or elongase enzyme systems [51]. In general, it would be useful to determine the genetic/biochemical mechanisms of PUFA synthesis in oil generating microbes in the development of SCO production systems [51, 96]. Of course, the fatty acids generated in the highest amount depend on the genetic makeup of each species. Fatty acid spectrum in oil generating yeasts is somewhat limited: most commonly found fatty acids include linoleic (18:2) and oleic (18:1) acids along with palmitoleic (16:1) or palmitic (16:0) acids [97]. Of the entire fatty acid content in fungi and microalgae, PUFAs are found to occur at a level of over 20 percent. Therefore, commercial attention has been sharpened specifically on those microalgae and fungi that generate desirable PUFAs in the highest quantity along with high triacylglycerol content.

Among oil-producing filamentous fungi, *M. circinelloides* is regarded as a vital organism for studying accumulation of lipids due to its capability to generate lipid rich in GLA [44]. GLA has several medicinal and health benefits, for instance treating and preventing diseases like cancers, diabetes, inflammatory disorders, and cardiovascular diseases [98–101]. Since 1980s, *M. circinelloides* has been commonly used as a model organism for lipid accumulation studies [102, 103]. In early studies, the proportion of GLA, linoleic acid, and oleic acid among fatty acids of *M. circinelloides* was reported to be 19%, 11%, and 38%, respectively [59, 104]. The major contributing factors during the GLA biosynthesis process in *M. circinelloides* were deciphered by Zhang et al. [105]. They carried out the homologous overexpression studies of two delta-6 (delta 6-2 and delta 6-1) and delta-12 desaturases to boost the GLA generation in *M. circinelloides*. During these studies, they found that GLA yield and GLA in total fatty acids were increased up to 33% and 38%, respectively, compared to the control strain. These investigations demonstrated that delta-6 desaturase plays a significant role in GLA generation in *M. circinelloides*. Perhaps the strain in which delta-6-1 desaturase was overexpressed has applications in the synthesis of microbial GLA [105]. Delta-12 desaturase overexpression increased the oleic acid conversion to linoleic acid which is then supposed to act as substrate for synthesis of GLA. Interestingly, instead of increase in GLA content, a decreasing trend was observed. The possible answer to this, might be that delta 12 desaturase isn’t the rate limiting enzyme for biosynthesis of GLA. Also, the over expression of delta-12 desaturase along with delta-6-1 desaturase showed less increase in GLA content compared to when delta-6-1 desaturase was overexpressed independently. It has been suggested that activity of delta-6-1 desaturase may have been repressed by delta-12 desaturase because of alteration in fluidity of membrane. Hence, future study may involve mutation of *delta-12 desaturase* gene to further enhance the GLA content.

GLA, when acted upon by the enzyme gamma-linolenic acid elongase (GLELO) or delta-6 elongase (D6E), results in the formation of an elongated product called Dihomo gamma-linolenic acid (DGLA, 20:3, n-6) [1]. Like GLA, DGLA also has nutritional value and medicinal applications. In recent times, DGLA has gained considerable biological attention because of its production of anti-inflammatory eicosanoids due to oxidative metabolism by lipoxygenases and cyclooxygenases [106]. DGLA generating cell factory was developed by overexpressing GLELO (D6E) gene in *M. circinelloides* CBS 277.49, producing up to 5.72% DGLA [107]. Previously, *Aspergillus oryzae* and *Saccharomyces cerevisiae* were metabolically engineered to produce DGLA [1, 108]. However, the concentration of DGLA in these microorganisms was observed to be very less. Engineered *M. circinelloides* strain showed the benefit of producing high DGLA concentration.

GLA also leads to the formation of Stearidonic acid (SDA) when acted upon by an enzyme called delta 15-desaturase [109]. SDA can be elongated to very long-chain length omega-3 PUFA with similar biological features, such as docosahexaenoic acid, eicosapentaenoic acid [110]. But SDA is more compliant to a variety of beverage and food applications as it is less prone to oxidation, hence more stable as compared to docosahexaenoic acid and eicosapentaenoic acid [111]. GLA is produced by *M. circinelloides;* hence it was used as a microbial chassis to produce SDA. To produce SDA, the delta-15 desaturase gene from *Mortierella alpina* was cloned and overexpressed *in M. circinelloides* CBS 277.49*,* leading to the formation of 5% SDA of the total fatty acids [111]. 13% SDA can be produced by a genetically engineered *Saccharomyces cerevisiae,* when supplemented with histidne [112, 113]. However engineered *M. circinelloides* strain produced SDA without any supplement. Nevertheless, utilization of this fungus to generate SDA industrially requires more research. It’s important to note here that all the research done to enhance GLA, DGLA and SDA content in *M. circinelloides* has been done in CBS 277.49, a low lipid producing strain. In future if WJ11, a high lipid producing strain is used, it may further enhance the PUFA content.

### Engineering of FAS, fatty acid beta-oxidation, and generation of MCFAs

As described by Hussain et al. [114], initiation of biosynthesis of fatty acids takes place according to the following reaction:

6 $${\text{Acetyl}} - {\text{CoA }} + {\text{ CO}}_{2} \xrightarrow{{{\text{Acetyl}} - {\text{CoA carboxylase}}}}{\text{Malonyl}} - {\text{CoA }}$$

Subsequently, acetyltransferase in FAS reaction centre, converts malonyl-CoA / acetyl-CoA into malonyl-ACP/acetyl-ACP, respectively. Phosphopantetheinyl transferase is involved in the activation of these ACPs. The malonyl-ACP is then decarboxylated and condensed with acetyl-ACP with the help of an enzyme ketosynthase. FAS complex domains including enoylreductase, ketoreductase and dehydratase aid the production of saturated acyl-ACP molecules by removing carbon double bonds and oxygen atoms. In the fungal FAS complex, ultimately decarboxylated malonyl-ACP molecules may be added to acyl-ACP molecules as a substrate, and this step lasts as long as malonyl/palmitoyl transacylase helps in the generation of long-chain fatty acids by catalyzing the termination step of the fatty acid synthesis. For increasing the chain length of fatty acids, thioesterase is an important metabolic regulator in plant and bacterial FAS complex as compared to fungal FAS [115–118]. Fatty acid profile of *M. circinelloides* indicated that it typically generates long-chain fatty acids (LCFAs), thereby making its industrial appeal displeasing [29, 57, 105, 119–123].

Medium-chain fatty acids (MCFAs), as well as their by-products having 8–12 carbon atoms chain length, are associated with the generation of green fuels [124]. Over the prior 10 years, as part of the nutritive diet plan, MCFAs have gained recognition as they can be utilized for the treatment/prevention of different disorders related to metabolism, including cardiovascular diseases, hypertension, hyperlipidemia, over-weight problems, type II diabetes, and atherosclerosis [125]. It has been observed that the amount of MCFAs grown from botanical resources is inadequate to satisfy the industrial needs [126, 127]. Thus, de novo biosynthesis of MCFAs from various lipogenic microorganisms has become the new focus of research [126–134].

Hussain et al. [114] metabolically engineered *M. circinelloides* M65 strain to create a heightened concentration of MCFAs (i.e., C8-C12). To actualize this objective, 4 discrete acyl carrier protein (ACP) thioesterase (TE)—coding genes having a choice of substrate for acyl ACP molecules with medium chain length were expressed in *M. circinelloides* M65, causing the development of fatty acid residues with the chain length of 8–12 carbons. The generation of a high amount of MCFAs was followed by a simultaneous decrease in the production of indigenous long-chain fatty acid residues, which indicated that the machinery of fatty acid biosynthesis (FASs) meddled with embedded TEs by channelizing acyl ACP molecules to MCFAs non-native development. It has also been reported that MCFAs are incorporated in several classes of lipids [114].

To improve MCFAs content in various microorganisms, several strategies have been employed so far. In this context, Hussain et al. [135] used a synergistic novel methodology for enhancing MCFA production in *M. circinelloides*. In wild-type fungal FAS, they integrated thioesterase protein from M65 (*M. circinelloides* uracil auxotroph) into MU758 (*M. circinelloides* leucine and uracil auxotroph) followed by modification of peroxisomal fatty acid β-oxidation pathway to avoid and maintain oxidation of MCFAs and LCFAs, respectively. During this modification, gene disruption of acyl-CoA oxidase (having a medium-chain acyl-CoAs preference of substrate) occurred.. Moreover, the acyl-CoA thioesterase gene was also disrupted to validate its role in the breakdown of fatty acids to free fatty acids and MCFAs transfer outside peroxisomes**.** It was concluded that high MCFAs amount could be generated with this combinative approach [135]. Some studies have modified the beta oxidation pathway (Fig. 2) in such a way that potential virulence in many strains of the fungi is significantly reduced [136–140]. However, further researches are desired for their verification. The future studies, to enhance MCFAs can be related to generation of recombinant strains with genetically engineered genes of beta oxidation pathway like *thiolase acetyl-synthetase, acyl-CoA oxidase* and *acyl-CoA thioesterase* [35, 124, 141]. Moreover, overexpression of various genes encoding various lipogenic enzymes can be done, which can elevate the total fatty acid content, thereby increasing MCFAs [92, 142–144].

## Versatile applications of *M. circinelloides*

*Mucor circinelloides* is deemed as a “microbial cell factory” because of its ability to generate microbial lipids, enzymes, essential amino acids, organic acids, ethanol, polyphenols, and food pigments [29, 78]. It is considered as a model organism to investigate carotenoids biosynthesis, especially beta carotene and astaxanthin. Carotenoids encompass several pigments existing in nature, having antioxidant activities [145] and medicinal applications [145, 146]. The utilization of *M. circinelloides* to study beta carotene regulation and biosynthesis began many years ago, revealing vital information about its recombinants and genes [147–150].

Studies carried out by our group [151] discovered that beta carotene is generated dominantly by *M. circinelloides* CBS 277.49 compared to *M. circinelloides* strain WJ11 [151]. Therefore, for further investigations, we [9] choose *M. circinelloides* CBS 277.49 strain for optimizing beta carotene production via response surface methodology. In our results, it was found that optimized media containing ketoconazole 150 mg/L, cerulenin 10 μg/mL, and the carbon–nitrogen ratio of 25 helped in the generation of beta carotene that was elevated by 157% [9]. *M. circinelloides* has also been investigated for the generation of a precious carotenoid called astaxanthin by the introduction of genes from *Paracoccus* sp. N81106 (formerly *Agrobacterium aurantiacum*) [28].

*Mucor circinelloides* can also generate natural antioxidants and nutraceuticals by the exploitation of its secondary metabolites and antioxidant sources [29]. During the investigations carried out by Hameed et al. [29], it was found that extracts of fungus displayed outstanding reducing capacity, chelation properties for metals, and scavenging of radicals in different assays like Ferric Reducing Antioxidant Power, Cupric Ion Reducing Antioxidant Capacity beta carotene, etc. The recovery of antioxidant yield was found to be highest in extracts from ethanol and hot water. It is mainly the phenolic content and condensed form of tannin of the extracts, which was found to be responsible for their antioxidant properties [29].

Although lipid and enzyme production constitutes to be the main application for *M. circinelloides*, some of the prospective utilizations other than these applications have been cited in the literature. *M. circinelloides* has been considered a potential ethanol producer, varying in aerobic and anaerobic conditions [152]. The biosynthetic mechanism of biofilms in fungi and microalgae has also been unfolded by utilizing *M. circinelloides* as a model organism [31, 153–157]. Applications of *M. circinelloides* have also been reported in the field of wastewater treatment [30] and bioremediation [31]. Its bioremediation potential is attributed to its capability of accumulating polyphosphates [158] and absorption of heavy metals [159, 160]. Various applications of *M. circinelloides* have been summarized in Table 4.

**Table 4:** Summary of applications using *M. circinelloides*

Strain used	Application	Study particularity	References
*M. circinelloides* CBS 277.49	Beta-carotene	Engineering of mevalonate pathway for improved beta carotene generation	[9]
*M. circinelloides* CBS 277.49	Astaxanthin	Investigating *M. circinelloides* for astaxanthin production	[28]
*M. circinelloides*WJ11 and CBS 277.49	Antioxidants and nutraceuticals	Investigating *M. circinelloides* for potential of antioxidants	[29]
*M. circinelloides* ATCC 1216B	Ethanol production	Ethanol generation using *M. circinelloides* via aerobic and anaerobic modes	[152]
*M. circinelloides* ATCC 1216B	Wastewater treatment	Recovery of phosphorus from wastewater of dairy manure	[30]
*M. circinelloides* UMN-B34; *Fusarium equiseti* (A11); *Fusarium lacertarum* (A13); *Nigrospora oryzae* (A16); *Altermaria alternate* (A20); *Fusarium equiseti* (B5); *Mucor hiemalis* (B7) and *Mortierella isabellina* (MI)	Biofilms	Utilizing fungi and algae strains for development of biofilms	[31]
*M. circinelloides*	Biosurfactant production	Investigation of *M. circinelloides* in Biosurfactant production using waste frying oil	[32]
*M. circinelloides* and *Trichoderma asperellum*	Phytoremediation	Cadmium and lead phytoremediation	[176]

## Conclusions and future prospects

In the areas of human health and biodiesel development, lipid bio-production is an interesting subject. *M. circinelloides* has been established as one of the most encouraging resources of various fatty acids for sustainable use. Extensive studies have been carried out regarding lipid accumulation metabolism in *M. circinelloides* because its oil acts as an important source for GLA which has important medicinal applications. The imbalance of nutrients involving the deficiency of nitrogen and excess of carbon actually triggers lipid accumulation in oleaginous fungi. Over the years, metabolic engineering of key enzymes and pathways like ME, G6PD, 6PGD, DGAT, FAS, PPP, AMPKs, citrate and malate transporters have taken place for building the oil-overproducing strains of *M. circinelloides*. However most of the metabolic engineering studies have taken place utilizing CBS 277.49, a low lipid producing strain of *M. circinelloides*. A new scope for further enhancement of lipid production via metabolic engineering can involve utilization of WJ11, a high lipid producing strain. Moreover, significant work has been carried out in the field of genomics, which has provided an important insight into genes that can be targeted in future to enhance the lipid yield. The most challenging obstacle for producing PUFAs using oleaginous *M. circinelloides* is to generate a large amount of PUFAs. In spite of recent achievements in the engineering of *M. circinelloides* for improved PUFA production, there is still considerable room for betterment. For example investigations into regulatory PUFA mechanism can be utilized to significantly enhance its yield.Future prospects for *M. circinelloides* can also involve a thorough investigation and screening of low cost substrates for its sustainable growth. Manipulation of signalling pathways and study of regulation mechanism of lipid accumulation by nutritional factors can also be helpful for improved lipid generation. For the purpose of producing competent microbial cell factories to generate lipid-based products for non-food and food applications, *M. circinelloides* as a model organism has become a powerful tool. Therefore, it is of great importance to harness the potential power of oleaginicity of *M. circinelloides* to as much extent as possible. Globally, seeking novel energy options to substitute petroleum has been a burning subject since the mid-1970s oil crisis. Biodiesel production has gained significant interest in the modern era because of several benefits over traditional energy supplies. We anticipate that, in upcoming years, there would be rapid progress in lipid metabolism research in *M. circinelloides,* and by employing the technology of metabolic engineering, lipid content would grow swiftly.

## Supplementary Information

**Additional file 1: Table S1.** Abbreviations of substrates and transporters in Fig. 2.

**Additional file 2: Table S2.** List of numbers denoting different enzymes in Fig. 2.

## Data Availability

Not applicable.
