# Supplementary material for: Mucor circinelloides: a model organism for oleaginous fungi and its potential applications in bioactive lipid production
Source: Microb Cell Fact. 2022 Feb 28;21:29. doi: 10.1186/s12934-022-01758-9 (PMC8883733; doi:10.1186/s12934-022-01758-9)
Supplement: Supplementary file 1 — Additional file 1: Table S1. Abbreviations of substrates and transporters in Fig. 2. [file 12934_2022_1758_MOESM1_ESM.docx]

**Table S1. Abbreviations of substrates, transporters and pathways in Fig. 2**

| **Abbreviations** | **Substrates/transporters/pathways** |
| --- | --- |
| Glu | glucose |
| G6P | glucose 6-phosphate |
| F6P | Fructose 6-phosphate |
| FBP | fructose 1,6-bisphosphate |
| G3P | glyceraldehyde 3-phosphate |
| 6PGL | 6-phosphogluconolactone |
| 6PG | 6-phosphogluconate |
| Ru5P | ribulose 5-phosphate |
| Ri5P | ribose 5-phosphate |
| GBP | glycerate 1,3-bisphosphate |
| 3PGA | 3-phosphoglyceric acid |
| 2PGA | 2-phosphoglyceric acid |
| PEP | phosphoenolpyruvate |
| Pyr | pyruvate |
| AcCoA | acetyl coenzyme A |
| Cit | citrate |
| Isocit | isocitrate |
| AKG | 2-ketoglutarate |
| SuCoA | succinyl coenzyme A |
| Suc | succinate |
| Fum | fumarate |
| Mal | malate |
| OAA | oxaloacetate |
| MaCoA | malonyl coenzyme A |
| AcAcCoA | acetoacetyl coenzyme A |
| HMGCoA | 3-hydroxy-3-methylglutaryl coenzyme A |
| MVA | mevalonate |
| MVA5P | mevalonate-5-phosphate |
| MVA5PP | mevalonate-5-pyrophosphate |
| IPP | 3-isopentenyl pyrophosphate |
| FPP | farnesyl pyrophosphate |
| GGPP | geranylgeranyl pyrophosphate |
| DHAP | dihydroxyacetonephosphate |
| Glyc3P | glycerol 3-phosphate |
| LysoPA | lysophosphatidic acid |
| PA | phosphatidic acid |
| DAG | diacylglycerol |
| TAG | triacylglycerol |
| FA | fatty acid |
| MT | malate transporter |
| CT | citrate transporter |
| TCT | tricarboxylate carrier |
| THC | transhydrogenase cycle |
| PPP | pentose phosphate pathway |
| TAG synthesis | triacylglycerol synthesis |
| TCA cycle | tricarboxylic acid cycle |
