# Supplementary material for: Mucor circinelloides: a model organism for oleaginous fungi and its potential applications in bioactive lipid production
Source: Microb Cell Fact. 2022 Feb 28;21:29. doi: 10.1186/s12934-022-01758-9 (PMC8883733; doi:10.1186/s12934-022-01758-9)
Supplement: Supplementary file 2 — Additional file 2: Table S2. List of numbers denoting different enzymes in Fig. 2. [file 12934_2022_1758_MOESM2_ESM.docx]

| Numbers | Enzymes |
| --- | --- |
| 1 | hexokinase |
| 2 | glucose-6-phosphate isomerase |
| 3 | phosphofructokinase |
| 4 | fructose-bisphosphate aldolase |
| 5 | glucose-6-phosphate dehydrogenase |
| 6 | 6-phosphogluconolactonase |
| 7 | 6-phosphogluconate dehydrogenase |
| 8 | ribose 5-phosphate isomerase A |
| 9 | transketolase |
| 10 | triose-phosphate isomerase |
| 11 | glycerol-3-phosphate dehydrogenase |
| 12 | glycerol-3-phosphate O-acyltransferase |
| 13 | 1-acylglycerol-3-phosphate acyltransferase |
| 14 | phosphatic acid phosphatase |
| 15 | diacylglycerol O-acyltransferase |
| 16 | triacylglycerol lipase |
| 17 | acyl-CoA synthetase |
| 18 | acyl-CoA dehydrogenase |
| 19 | enoyl-CoA hydratase |
| 20 | 3-hydroxyacyl-CoA dehyrogenase |
| 21 | 3-ketoacyl-CoA thiolase, mitochondrial |
| 22 | glyceraldehyde 3-phosphate dehydrogenase |
| 23 | phosphoglycerate kinase |
| 24 | phosphoglycerate mutase |
| 25 | enolase |
| 26 | pyruvate kinase |
| 27 | pyruvate carboxylase |
| 28 | malate dehydrogenase |
| 29 | malic enzyme, cytoplasmic |
| 30 (i) | pyruvate dehydrogenase |
| 30 (ii) | dihydrolipoamide acetyltransferase |
| 31 | citrate synthase |
| 32 | aconitate |
| 33 | isocitrate dehydrogenase |
| 34 (i) | 2-ketoglutarate dehydrogenase |
| 34 (ii) | dihydrolipoamide succinyltransferase |
| 35 | succinyl-CoA ligase |
| 36 | succinate dehydrogenase |
| 37 | fumarate hydratase |
| 38 | malic enzyme, mitochondrial |
| 39 | aldehyde dehydrogenase |
| 40 | acetyl-CoA synthetase |
| 41 | ATP-citrate lyase |
| 42 | acetyl-CoA carboxylase |
| 43 | acetyl-CoA C-acetyltransferase |
| 44 | hydroxymethylglutaryl-CoA synthase |
| 45 | hydroxymethylglutaryl-CoA reductase |
| 46 | mevalonate kinase |
| 47 | phosphomevalonate kinase |
| 48 | diphosphomevalonate decarboxylase |
| 49 | geranylgeranyl pyrophosphate synthases |

**Table S2. List of numbers denoting different enzymes in Fig. 2.**
